# Patient and Provider Experiences With Virtual Care in a Large, Ambulatory Care Hospital in Ontario, Canada During the COVID-19 Pandemic: Observational Study

**DOI:** 10.2196/38604

**Published:** 2022-10-25

**Authors:** Cherry Chu, Dhruv Nayyar, Onil Bhattacharyya, Danielle Martin, Payal Agarwal, Geetha Mukerji

**Affiliations:** 1 Women's College Hospital Institute for Health System Solutions and Virtual Care Toronto, ON Canada; 2 Institute of Health Policy, Management and Evaluation Dalla Lana School of Public Health University of Toronto Toronto, ON Canada; 3 Temerty Faculty of Medicine University of Toronto Toronto, ON Canada; 4 Women’s College Hospital Toronto, ON Canada; 5 Department of Family and Community Medicine University of Toronto Toronto, ON Canada

**Keywords:** virtual care, telehealth, COVID-19 pandemic, patient experience, provider experience, virtual, telemedicine, COVID-19, ethnicity, social factors, experience

## Abstract

**Background:**

Virtual care use increased during the COVID-19 pandemic. The impact of that shift on patient and provider experiences is unclear.

**Objective:**

We evaluated patient and provider experiences with virtual visits across an academic, ambulatory hospital in Toronto, Canada and assessed predictors of positive experience with virtual care.

**Methods:**

Survey data were analyzed from consenting patients who attended at least one virtual visit (video or telephone) and from consenting providers who delivered at least one virtual visit. Distributions for demographic variables and responses to survey questions are reported, with statistical significance assessed using chi-square tests and t tests. Ordinal logistic regression analysis was used to identify any patient predictors of responses.

**Results:**

During the study period, 253 patients (mean age 45.1, SD 15.6 years) completed 517 video visit surveys, and 147 patients (mean age 41.6, SD 16.4 years) completed 209 telephone visit surveys. A total of 75 and 94 providers completed the survey in June 2020 and June 2021, respectively. On a scale from 1 to 10 regarding likelihood to recommend virtual care to others, fewer providers rated a score of 8 or above compared with patients (providers: 62/94, 66% for video and 49/94, 52% for telephone; patients: 415/517, 80% for video and 150/209, 72% for telephone). Patients of non-White ethnicity had lower odds of rating a high score of 9 or 10 compared with White patients (odds ratio 0.52, 95% CI 0.28-0.99).

**Conclusions:**

Patient experiences with virtual care were generally positive, but provider experiences were less so. Findings suggest potential differences in patient experience by ethnicity, warranting further investigation into equity concerns with virtual care.

## Introduction

The COVID-19 pandemic has accelerated virtual care use in many jurisdictions, stemming from the need for physical distancing, preservation of personal protective equipment, and the desire to adhere to public health guidance [1-3]. In the province of Ontario, Canada, virtual care adoption was low prior to the COVID-19 pandemic, in large part due to restrictive reimbursement policies [4]. The onset of the pandemic led to the introduction of temporary billing codes allowing for reimbursement of various modalities of virtual care, including videoconferencing across a wide range of platforms as well as telephone visits [5]. During this global crisis, virtual care use in Ontario increased significantly, from 1.6% of total ambulatory visits in the second quarter of 2019 to 70.6% in the second quarter of 2020 [6].

Many studies have been published on patient or provider experiences with virtual care before and during the pandemic [7-12]. Some studies have found positive experiences with virtual visits due to reasons such as convenience and travel time avoided [13-15], while other studies have reported that patients and providers did not find the quality of virtual visits to be better than in-person visits [16]. However, to our knowledge, the literature on patient and provider experiences with virtual care has been mostly limited to small-scale studies localized to a specific clinical program. Furthermore, few have attempted to address potential equity considerations that might contribute to differences in patient experience [17,18]. As virtual care becomes more prevalent, so does the potential issue of the digital divide, in which patients of marginalized populations, such as older age, lower health literacy, non-White ethnic backgrounds, or lower income, may have worse access to or experiences with virtual health services compared with others for reasons such as lack of access to technology or resources in general, discrimination, and limited digital health literacy [19,20].

At Women’s College Hospital (WCH), an academic ambulatory hospital in Toronto, Ontario, Canada, virtual care adoption accelerated during the COVID-19 pandemic. In-person ambulatory visits throughout the hospital were largely replaced with video or telephone appointments. In this study, our objective was to use data from WCH during the pandemic to describe patient and provider experiences with virtual care across various clinical areas and to identify demographic characteristics associated with patient experience.

## Methods

### Context

WCH is an ambulatory, academic facility in Toronto, Canada and is fully affiliated with the University of Toronto. From an administrative perspective, virtual visits are scheduled almost identically to in-person visits, the only difference being the visit type used. For video visits specifically, the electronic medical record is able to automatically create video encounters with the use of an existing platform (ie, Zoom, a video conferencing service that can be licensed for secure use for health care purposes) when specific video visit types are used. When booking a phone visit, it is clearly identified to clinicians that the visit is to take place by phone. The hospital provided resources on its website, notifying patients of the option of video virtual care and its availability as well as how to use it, including training videos and guides. Otherwise, patients would have been presented with the option during phone calls with administrative staff or notified of a virtual video or phone visit within the appointment notification letter they received. Clinicians were encouraged to curate their environment prior to conducting virtual visits, especially by video. In many instances, clinicians continued to work within the clinic when conducting virtual care, though several clinics or departments worked mostly or entirely from home. Regardless, creating private space and ensuring the use of appropriate technology were encouraged. Clinicians were also encouraged to collect appropriate identifying information from patients, much as would occur during check-in during an in-person visit. On the patient end, the resources provided to them motivated them to treat virtual encounters much as they would in-person encounters, including ensuring their own private space, being in a well-lit area, and minimizing background distractions.

### Ethical Approval

This study received ethics approval from the WCH Research Ethics Board (REB # 2019-0191-E).

### Data Sources and Population

Data were collected from surveys administered to consenting patients who attended at least one virtual visit (video or telephone) at any clinic within WCH who consented via digital consent to be sent a patient experience survey after their visit. Patients had to be registered in the patient portal to receive the digital consent via the electronic medical record. Patients were offered the opportunity to complete the survey after every virtual visit attended. We analyzed patient experience survey data for video visits from May 2020 to May 2021 (253 patients and 517 responses). As survey deployment for telephone visits was delayed due to staff shortages in the second pandemic wave, we analyzed data for telephone visits since survey launch from October 2020 to May 2021 (147 patients and 209 responses). Provider experience surveys were administered twice, in June 2020 and June 2021, for those who delivered at least one virtual visit at any clinic within the hospital. Both patient and provider surveys included questions regarding demographic characteristics and satisfaction with virtual care, with opportunities for written feedback. Patient and provider survey questions can be found in Multimedia Appendix 1.

### Statistical Analysis

Descriptive statistics were used to summarize demographic characteristics and responses to patient experience questions among all patients and new patients (defined as those who had an initial visit with a clinic during the study period) who attended at least one virtual visit at the hospital. The following demographic characteristics were self-identified through survey responses: age, gender, ethnicity, household income, and English-language proficiency. Overall marginalization was determined using the Ontario marginalization index (ON-Marg) [21], which was linked to patient postal codes. ON-Marg is a tool that measures deprivation on multiple levels, including economic, ethnoracial, age-based, and social marginalization. Chi-square tests, Fischer exact tests, and *t* tests were performed as applicable to compare demographic characteristics and responses to various questions regarding patient experience (eg, helpfulness of the virtual visit, likelihood of recommending virtual visits to a friend) between patients who attended video appointments and patients who attended telephone appointments.

The net promoter score, a metric used to measure a client’s willingness to recommend a company’s product or services [22], was also calculated for video and telephone visits. As the net promoter score can range from –100% to +100%, any score greater than 0% can be considered a desirable score [23]. Ordinal logistic regression analysis was performed on all survey responses to identify any patient demographic variables that may predict their response to select questions on likelihood to recommend virtual visits and perceived helpfulness of the virtual visits. Findings from the regression model compare the odds of choosing an answer in the highest category compared with the other 2 categories (eg, rating of 9-10 compared with ratings of 1-6 and 7-8). Only patients with complete responses to all relevant survey questions were included in the models. Provider ratings to various survey questions on their experience, including the perceived quality of the virtual visit compared with an in-person visit, the amount of time and effort required to conduct the virtual visit, and others, were compared descriptively between June 2020 and June 2021.

## Results

### Patient Experience

Among all virtual visits for patients registered to the patient portal during the study period, the proportion of individuals who consented to be sent a survey was 1057 of 1872 (56.5%) for video visits and 259 of 358 (72.3%) for phone visits. Among those who consented, 517 of 1057 (48.9%) video visit surveys and 209 of 259 (80.7%) phone visit surveys were completed. Baseline characteristics of all patients who responded to at least one video or telephone survey are reported in Table 1. A total of 253 unique patients completed 517 video surveys, while 147 unique patients completed 209 telephone surveys. There were more women (130/147, 88.4% vs 195/253, 77.1%; *P*=.005) and older patients (mean age 45.1, SD 15.6 years vs mean 41.6, SD 16.4 years; *P*=.04) who completed telephone visit surveys compared with video visit surveys. Among survey respondents who were new patients, telephone users were also older than video users (mean age 50.5, SD 17.5 years vs mean 41.3, SD 15.3 years; *P*=.02; Table S1 in Multimedia Appendix 2). The top clinical departments through which patients attended the virtual visits are listed in Tables S2 and S3 in Multimedia Appendix 2.

Table 2 reports patient responses regarding their experience with virtual care. Most patients found their virtual visit to be very helpful for their health issue (417/517, 80.7% for video and 154/209, 73.7% for telephone). When asked what they would have done if a virtual visit with the doctor was not available, most selected “I would not have sought care at that time” (198/517, 38.3% of video users and 43/209, 20.6% of telephone users), “Scheduled an in-person visit with this doctor” (192/517, 37.1% of video users and 99/209, 47.4% of telephone users), or “See/talk to my family doctor” (145/517, 28.1% of video users and 58/209, 27.8% of telephone users). On a scale of 1 to 10 regarding likelihood to recommend virtual care to a friend, most patients responded with a rating of 8 or above (415/517, 80.3% of video users and 150/209, 71.8% of telephone users). The net promoter score for video visits was higher than that for telephone visits (60.2% vs 40.4%). Findings were similar when only considering new patients (Table S4 in Multimedia Appendix 2)**.** However, the difference in net promoter scores between video and telephone visits was greater for new patients (51.8% for video vs 15.0% for telephone). The majority of patients (405/517, 78.3% for video and 146/209, 69.9% for telephone) preferred to have the option of virtual visits after COVID-19.

We report results for the ordinal logistic regression models in Table 3. In model 1, 255 patients with complete responses were included, and 268 patients were included in model 2. From model 1, ethnicity and age group were significant predictors of the likelihood of recommending video or phone visits to a friend. Specifically, patients who were non-White had lower odds of rating a high score of 9 or 10 compared with White patients (odds ratio [OR] 0.52, 95% CI 0.28-0.99), and patients aged 50 years to 59 years had lower odds of rating a high score of 9 or 10 compared with patients aged 30 years to 39 years (OR 0.26, 95% CI 0.11-0.64). None of the independent variables (ethnicity, family income, overall marginalization, gender, and age) assessed in model 2 appeared to be significant predictors of how helpful the virtual visit was in addressing the patient’s health issue.

**Table 1:** Baseline patient characteristics of all video and phone survey respondents.

Characteristic			Video visit survey respondents (n=253)^a^		Video visit survey respondents among nonmissing respondents, n (%)		Phone visit survey respondents (n=147)^b^		Phone visit survey respondents among nonmissing respondents, n (%)		*P* value^c^
**Gender, n (%)**											
	Female	195 (77.1)		195 (77.1)		130 (88.4)		130 (88.4)		.005	
	Male	58 (22.9)		58 (22.9)		17 (11.6)		17 (11.6)			
Age (years), mean (SD)			41.6 (16.4)		N/A^d^		45.1 (15.6)		N/A		.04
**Total family income (CAD) in previous year^e^, n (%)**											
	0 to 29,999	9 (3.5)		9 (8.3)^f^		8 (5.4)		8 (5.5)^g^		.82	
	30,000 to 59,999	10 (4.0)		10 (9.3)^f^		12 (8.2)		12 (8.2)^g^			
	60,000 to 89,999	12 (4.7)		12 (11.1)^f^		20 (13.6)		20 (13.7)^g^			
	90,000 to 119,000	15 (5.9)		15 (13.9)^f^		17 (11.6)		17 (11.6)^g^			
	120,000 to 149,000	4 (1.6)		4 (3.7)^f^		10 (6.8)		10 (6.8)^g^			
	150,000 or more	26 (10.3)		26 (24.1)^f^		27 (18.4)		27 (18.5)^g^			
	Do not know	5 (2.0)		5 (4.6)^f^		8 (5.4)		8 (5.5)^g^			
	Prefer not to answer	27 (10.7)		27 (25.0)^f^		44 (29.9)		44 (30.1)^g^			
	Missing	145 (57.3)		N/A		1 (0.7)		N/A			
**Ethnicity^h^, n (%)**											
	White	82 (32.4)		82 (73.2)^i^		102 (69.4)		102 (70.8)^j^		.54	
	Asian	9 (3.6)		9 (8.0)^i^		19 (12.9)		19 (13.2)^j^			
	Black	4 (1.6)		4(3.6)^i^		4 (2.7)		4 (2.8)^j^			
	Latin American	5 (2.0)		5 (4.5)^i^		2 (1.4)		2 (1.4)^j^			
	Indigenous	1 (0.4)		1 (0.9)^i^		1 (0.7)		1 (0.7)^j^			
	Middle Eastern	2 (0.8)		2 (1.8)^i^		2 (1.4)		2 (1.4)^j^			
	Mixed heritage/other(s)	3 (1.2)		3 (2.7)^i^		8 (5.4)		8 (5.6)^j^			
	Prefer not to answer	6 (2.4)		6 (5.4)^i^		6 (4.1)		6 (4.2)^j^			
	Missing	141 (55.7)		N/A		3 (2.0)		N/A			
**English-speaking ability, n (%)**											
	Very well	108 (42.7)		108 (96.4)^i^		139 (94.6)		139 (94.6)^k^		.77	
	Well	3 (1.2)		3 (2.7)^i^		7 (4.8)		7 (4.8)^k^			
	Not well	1 (0.4)		1 (0.9)^i^		1 (0.7)		1 (0.7)^k^			
	Missing	141 (55.7)		N/A		0 (0)		N/A			
**Ontario marginalization index, n (%)**											
	Marginalized	36 (14.2)		36 (14.9)^l^		16 (10.9)		16 (11.6)^m^		.37	
	Not marginalized	206 (81.4)		206 (85.1)^l^		122 (83.0)		122 (88.4)^m^			
	Missing	11 (4.4)		N/A		9 (6.1)		N/A			

^a^Number of unique patients who responded of 517 video survey responses received. The same patient may be counted multiple times.

^b^Number of unique patients who responded of 209 phone survey responses received. The same patient may be counted multiple times.

^c^*P* value compares the distribution of demographic variables between video and telephone groups.

^d^N/A: not applicable.

^e^*P* value compares <$90,000 with ≥$90,000.

^f^n=108.

^g^n=146.

^h^*P* value compares White with non-White.

^i^n=112.

^j^n=144.

^k^n=147.

^l^n=242.

^m^n=138.

**Table 2:** Video and phone survey responses.

Question		Video visit responses (n=517)	Phone visit responses (n=209)	*P* value^a^
**To what degree did the video or phone visit help you with the health issue for which you needed the appointment?, n (%)**				
	Not at all helpful	3 (0.6)	1 (0.5)	.14
	Not helpful	3 (0.6)	2 (1.0)	
	Neutral	17 (3.3)	15 (7.2)	
	Somewhat helpful	75 (14.5)	35 (16.8)	
	Very helpful	417 (80.7)	154 (73.7)	
	Missing	2 (0.4)	2 (1.0)	
**What would you have done if you were not able to see your doctor through a video or phone visit?^b^, n (%)**				
	Walk-in clinic	20 (3.9)	2 (1.0)	<.001
	Emergency department	32 (6.2)	6 (2.9)	
	See/talk to my family doctor	145 (28.1)	58 (27.8)	
	Scheduled an in-person visit with this doctor	192 (37.1)	99 (47.4)	
	I would not have sought care at that time	198 (38.3)	43 (20.6)	
	Missing	0 (0)	1 (0.5)	
**How likely are you to recommend video or phone visits to a friend on a scale of 1 to 10? (1 = would not recommend and 10 = would highly recommend), n (%)**				
	1	1 (0.2)	2 (1.0)	.02
	2	1 (0.2)	4 (1.9)	
	3	3 (0.6)	0 (0.0)	
	4	3 (0.6)	2 (1.0)	
	5	20 (3.9)	13 (6.2)	
	6	17 (3.3)	11 (5.3)	
	7	42 (8.1)	16 (7.7)	
	8	68 (13.2)	38 (18.2)	
	9	78 (15.1)	26 (12.4)	
	10	269 (52.0)	86 (41.2)	
	Missing	15 (2.9)	11 (5.3)	
Net promoter score, %		60.2	40.4	N/A^c^
**Would you like the option to continue having virtual visits with your health care providers after COVID-19?, n (%)**				
	No	19 (3.7%)	19 (9.1%)	.004
	Not sure	89 (17.2%)	44 (21.1%)	
	Yes	405 (78.3%)	146 (69.9%)	
	Missing	4 (0.8%)	0 (0%)	

^a^*P* value compares the distribution of survey responses between video and telephone groups.

^b^Multiselect question for video visit survey.

^c^N/A: not applicable.

**Table 3:** Ordinal logistic regression analysis results.

Variable			Model 1: How likely are you to recommend video or phone visits to a friend on a scale of 1 to 10? (1 = would not recommend and 10 = would highly recommend)^a^ (n=255)				Model 2: To what degree did the video or phone visit help you with the health issue for which you needed the appointment?^b^ (n=268)		
			OR^c^ (95% CI)		*P* value		OR (95% CI)		*P* value
**Ethnicity** **(reference: White)**									
	Non-White	0.52 (0.28-0.99)		.047		0.87 (0.42-1.77)		.69	
	Prefer not to answer	1.19 (0.35-4.12)		.78		1.33 (0.33-5.39)		.69	
**Family income** **(reference: $150,000 CAD or more)**									
	0 to 29,999	3.28 (0.63-17.09)		.16		1.56 (0.28-8.61)		.61	
	30,000 to 59,999	0.62 (0.21-1.84)		.39		0.51 (0.15-1.72)		.28	
	60,000 to 89,999	0.52 (0.19-1.45)		.21		0.74 (0.24-2.33)		.61	
	90,000 to 119,000	0.67 (0.25-1.76)		.42		0.38 (0.13-1.09)		.07	
	120,000 to 149,999	1.16 (0.26-5.20)		.84		0.42 (0.10-1.81)		.25	
	Do not know	1.53 (0.44-5.29)		.50		0.60 (0.17-2.14)		.44	
	Prefer not to answer	0.52 (0.23-1.15)		.11		0.65 (0.26-1.64)		.36	
**Gender** **(reference: Male)**									
	Female	0.99 (0.48-2.04)		.98		1.70 (0.81-3.58)		.16	
**Overall marginalization** **(reference: Not marginalized)**									
	Marginalized	1.87 (0.78-4.49)		.16		2.15 (0.78-5.89)		.14	
**Age group** **(years; reference: 30-39)**									
	0-18	0.32 (0.05-1.92)		.21		1.29 (0.15-11.28)		.82	
	19-29	0.63 (0.26-1.54)		.31		0.76 (0.29-1.99)		.57	
	40-49	0.73 (0.34-1.58)		.42		0.72 (0.31-1.71)		.46	
	50-59	0.26 (0.11-0.64)		.003		0.53 (0.20-1.40)		.20	
	60-69	1.19 (0.39-3.63)		.76		1.30 (0.39-4.35)		.67	
	≥70	0.95 (0.33-2.75)		.92		0.83 (0.28-2.41)		.73	

^a^Outcome categories: “1-6,” “7-8,” “9-10.”

^b^Outcome categories: “not helpful or neutral,” “somewhat helpful,” “very helpful.”

^c^OR: odds ratio.

### Provider Experience

A total of 75 providers completed the survey in June 2020, and 94 providers completed the survey in June 2021 (Table 4). The top 3 survey respondents among providers in 2020 were physicians (47/75, 63%), social workers (7/75, 9%), and psychotherapists (6/75, 8%), while the top 3 provider survey respondents in 2021 were physicians (48/94, 51%), social workers (9/94, 10%), and physiotherapists (8/94, 9%). In both 2020 and 2021, most providers who delivered virtual visits had been practicing for 10 or more years (40/75, 53% in 2020 and 61/94, 65% in 2021).

Responses to the provider experience surveys are shown in Table 5. When asked whether the quality of the virtual visit was similar to that of an in-person visit, 13% (10/75) selected agree or strongly agree in 2020, compared with 28% (26/94) in 2021. In 2020, 67% (50/75) of providers felt that video visits enabled them to sufficiently address their patient’s clinical need compared with 70% (66/94) in 2021. Most providers planned to continue using video visits after the need for physical distancing decreased (53/75, 71% in 2020 and 69/94, 73% in 2021). When asked to rate on a scale of 1 to 10 their likelihood of recommending other providers to do virtual visits for patients, most providers rated a score of 8 or above in 2020 (47/75, 63% for video and 46/75, 61% for telephone) and in 2021 (62/94, 66% for video and 49/94, 52% for telephone). The net promoter scores for video visits were 17.8% in 2020 and 30.4% in 2021, while the net promoter scores for telephone visits were 19.2% in 2020 and 1.1% in 2021.

**Table 4:** Baseline characteristics of providers who delivered at least one virtual visit.

Variable		June 2020 (n=75), n (%)	June 2021 (n=94), n (%)
**Provider type**			
	Dietitian	0 (0)	2 (2)
	Kinesiologist	2 (3)	2 (2)
	Nurse	2 (3)	7 (8)
	Nurse practitioner	2 (3)	5 (5)
	Occupational therapist	2 (3)	2 (2)
	Pharmacist	0 (0)	1 (1)
	Physician	47 (63)	48 (51)
	Physiotherapist	4 (5)	8 (9)
	Psychologist	1 (1)	2 (2)
	Psychotherapist	6 (8)	5 (5)
	Social service worker	1 (1)	3 (3)
	Social worker	7 (9)	9 (10)
	Other	1 (1)	0 (0)
**Years in practice**			
	1-2	9 (12)	9 (10)
	3-5	7 (9)	10 (11)
	6-7	12 (16)	6 (6)
	8-9	6 (8)	7 (8)
	≥10	40 (53)	61 (65)
	Missing	1 (1)	1 (1)

**Table 5:** Provider experience survey responses.

Question			June 2020 (n=75)		June 2021 (n=94)
**The quality of examination virtually was similar to an in-person exam., n (%)**					
	Strongly disagree	11 (15)		11 (12)	
	Disagree	30 (40)		21 (22)	
	Neutral	11 (15)		17 (18)	
	Agree	9 (12)		20 (21)	
	Strongly agree	1 (1)		6 (6)	
	Missing	13 (17)		19 (20)	
**The video visit enabled me to sufficiently address the patient’s clinical need., n (%)**					
	Strongly disagree	0 (0)		2 (2)	
	Disagree	7 (9)		3 (3)	
	Neutral	12 (16)		15 (16)	
	Agree	41 (55)		38 (40)	
	Strongly agree	9 (12)		28 (30)	
	Missing	6 (8)		8 (9)	
**I spent the same amount of time on the video visit as I would have for an in-person visit., n (%)**					
	Strongly disagree	4 (5)		3 (3)	
	Disagree	16 (21)		17 (18)	
	Neutral	7 (9)		6 (6)	
	Agree	28 (37)		35 (37)	
	Strongly agree	13 (17)		24 (26)	
	Missing	7 (9)		9 (10)	
**I spent the same amount of effort on the video visit as I would have for an in-person visit., n (%)**					
	Strongly disagree	4 (5)		6 (6)	
	Disagree	29 (39)		22 (23)	
	Neutral	11 (15)		12 (13)	
	Agree	17 (23)		27 (29)	
	Strongly agree	8 (11)		16 (17)	
	Missing	6 (8)		11 (12)	
**I feel I can deliver the same quality care using video visits as in person., n (%)**					
	Strongly disagree	1 (1)		9 (10)	
	Disagree	23 (31)		12 (13)	
	Neutral	23 (31)		19 (20)	
	Agree	24 (32)		35 (37)	
	Strongly agree	2 (3)		18 (19)	
	Missing	2 (3)		1 (1)	
**I feel I can deliver the same quality care using phone visits as in person., n (%)**					
	Strongly disagree	2 (3)		9 (10)	
	Disagree	26 (35)		22 (23)	
	Neutral	23 (31)		21 (22)	
	Agree	22 (29)		27 (29)	
	Strongly agree	2 (3)		15 (16)	
	Missing	0 (0)		0 (0)	
**I plan to continue using video visits after the need for physical distancing decreases., n (%)**					
	Strongly disagree	0 (0)		4 (4)	
	Disagree	6 (8)		4 (4)	
	Neutral	16 (21)		15 (16)	
	Agree	31 (41)		24 (26)	
	Strongly agree	22 (29)		45 (48)	
	Missing	0 (0)		2 (2)	
**On a scale of 1 to 10, how likely are you to recommend other providers like yourself do video visits for patients?, n (%)**					
	1	1 (1)		2 (2)	
	2	0 (0)		1 (1)	
	3	1 (1)		1 (1)	
	4	2 (3)		2 (2)	
	5	3 (4)		5 (5)	
	6	4 (5)		6 (6)	
	7	15 (20)		13 (14)	
	8	23 (31)		17 (18)	
	9	10 (13)		15 (16)	
	10	14 (19)		30 (32)	
	Missing	2 (3)		2 (2)	
Net promoter score, %			17.8		30.4
**On a scale of 1 to 10, how likely are you to recommend other providers like yourself do phone visits for patients?, n (%)**					
	1	0 (0)		3 (3)	
	2	1 (1)		3 (3)	
	3	0 (0)		3 (3)	
	4	0 (0)		3 (3)	
	5	4 (5)		12 (13)	
	6	7 (9)		10 (11)	
	7	15 (20)		11 (12)	
	8	20 (27)		14 (15)	
	9	11 (15)		9 (10)	
	10	15 (20)		26 (28)	
	Missing	2 (3)		0 (0%)	
Net promoter score, %			19.2		1.1

## Discussion

### Principal Findings

This study describes the patient and provider experiences with virtual visits during the COVID-19 pandemic across an academic ambulatory hospital in Toronto, Canada. Feedback for virtual visits was generally positive among patients. Video visits were the preferred modality over telephone among many patients, particularly for those who were new patients. However, we found that patients of non-White background were less likely to recommend virtual visits compared with those of White background. Provider experiences with virtual visits were less positive compared with those of their patients, but there was a general improvement in provider feedback from 2020 to 2021.

### Comparison With Prior Work

Most patients found that their virtual visit was helpful in addressing their health issue and rated a high score when asked to what degree they would recommend virtual visits to a friend. However, a higher proportion of patients reported video visits to be “very helpful” compared with telephone visits. Similarly, the net promoter score was much higher for video visits compared with telephone visits. In our study, patients who completed telephone visit surveys were generally female and older in age compared with patients who completed video visits. In the literature, older patients are less likely to engage in virtual care than in in-person care and even less likely to choose video than telephone [24,25].

In our study, several patients indicated in the open-ended questions that they would prefer video over telephone due to the ability to see the provider and observe facial expressions and body language. Other studies have also cited the benefit of increased human connection that accompanies video platforms [26]. The difference in net promoter scores was even greater among new patients, with video visits reaching a significantly higher score compared with telephone visits. Video visits enable patients to see their provider, which supports the development of a patient-provider relationship especially for an initial encounter. These results contrast with findings from a systematic review that reported no significant differences in patient satisfaction between video and telephone visits, but they did not stratify initial versus follow-up encounters [27]. This may also be attributed to our sample consisting of younger individuals. It is likely that younger patients prefer video visits more so than older patients who may prefer telephone visits due to ease of access [28].

Despite the generally positive feedback for virtual visits, a small proportion of patients did not find the visit helpful or rated a low recommendation score. The open-ended responses suggest that some patients were unhappy with the delay in their appointment start time and the lack of communication from the clinic in such cases. Published studies have cited other patient criticisms of virtual visits such as technical issues with connection and quality of the call [29], a lack of privacy at home when attending virtual visits [30], and a preference for in-person visits for certain physical health issues or to build a relationship with their provider [31]. Overall, it appears that many patients had positive experiences with their virtual visit(s), with several citing reasons such as convenience and that they were able to save time and money [13].

Findings from the regression model indicate that non-White patients were less likely to recommend virtual visits to a friend compared with White patients. Mixed findings are reported in the literature, with several studies showing no significant differences in patient experience with virtual care by ethnicity [32-34], while others have shown that patients of non-White backgrounds are more likely to have lower satisfaction with virtual visits compared with their White counterparts [17,35]. Upon analysis of the average recommendation scores for video versus telephone visits by ethnicity, shown in Table S5 in Multimedia Appendix 2, Asian and Black patients had similar, if not better, scores than White patients; however, ratings were generally lower for the other ethnic groups. Reasons for this disparity remain unclear and should be investigated in future work. Among all age groups of interest, only patients aged 50 years to 59 years were found to be less likely to recommend virtual visits than patients aged 30 years to 39 years (the reference group). This may be explained by older patients’ preferences toward in-person care or the technological barriers they may encounter with virtual visits [36,37]; however, this association did not persist in the older age groups (those older than 60 years) for reasons unknown. We do note that there were no significant differences in experience found for the other demographic variables assessed (family income, overall marginalization, sex, and most age groups), which may be a positive sign that the delivery of virtual visits may have helped to bridge the gap in equitable health care access in certain ways; for example, lower income patients may find it easier to attend a virtual visit than request time off work to attend in-person care, or older patients with mobility issues may find it easier to attend a virtual visit than an in-person visit.

Our findings are consistent with other studies that found lower satisfaction with virtual care among health care providers compared with patients [38]. Our findings show that the net promoter scores were lower for both video and telephone visits when rated by providers than by patients. Open-ended responses from the provider surveys suggest that many providers felt that they needed to provide a physical examination to adequately address their patient’s health needs, similar to findings from the available literature [39]. Other providers felt that the quality of the virtual visit was lower than that of an in-person visit. Several also cited technical issues, particularly with video visits, as a deterrent for virtual care. Studies examining provider experience with virtual care reported similar reasons for provider dissatisfaction [40], with less than one-half of providers preferring virtual over in-person care [41].

From a provider standpoint, there appeared to be an increase in positive feedback for virtual visits across most survey questions from 2020 to 2021, including quality of virtual visit, time and effort spent on virtual visit, and preference to use virtual care after the pandemic. This may be because, as providers had more experience with virtual care, their self-efficacy may have improved. Another explanation is that the proportion of all visits that were virtual was higher in 2020 than in 2021, so the appropriateness of virtual care for the visit reason was likely also higher in 2021. Several studies have reported that clinicians have a positive outlook on virtual visits [13], particularly within the mental health field [39,42] in which physical examinations play a lesser role in clinical practice compared with other specialties. However, we note that the providers’ net promoter score for telephone visits decreased significantly from 2020 to 2021, while scores for video visits increased. A possible explanation supported by open-text portions of the survey is that providers may have been more comfortable and proficient with providing video visits and preferred the face-to-face connection that can be missing from telephone communication, but this merits further exploration.

### Strengths and Limitations

A strength of this study is that it provides the patient and provider perspectives on virtual care in a large ambulatory hospital setting with responses across many clinical specialties and programs. Our findings also offer insights into both patient and provider experiences and into some of the demographic differences in experience with virtual care to identify potential equity issues. Nonetheless, our study does have several limitations. The overall response rate for demographic questions in the video survey was lower than anticipated due to a technical error in survey deployment. Furthermore, despite our equity focus, there are limitations in the demographic insights that can be gleaned. First, although we captured several important demographic variables in this study, we were unable to assess the association between patient experience and other potentially relevant characteristics, such as education level, employment, and immigration status. The low proportion of patients who consented to receive surveys and the fact that surveys could only be sent to patients who had a valid email address and were registered on the portal system would have limited responses, including from certain marginalized and underserviced groups. We also acknowledge the possibility that patients who have fewer positive experiences with virtual visits may be less inclined to complete the survey, which would potentially bias the findings to be more positive*.* However, our analysis was still able to detect a difference in experience among patients of ethnic minorities. Finally, an electronic survey does not offer a deep understanding of experience, particularly among marginalized groups, as opportunities for feedback is limited and patients may not feel as comfortable sharing their thoughts on the platform due to confidentiality concerns. Future studies of patient experiences with virtual care should include interviews or focus groups with patients from underserved communities. We also surveyed patients seen in specialty clinics within an ambulatory care hospital and did not include primary care patient surveys. Last, these findings reflect the experiences of patients and providers in a single institution within a universal health care system and therefore may not be generalizable to other settings.

### Conclusions

This study summarizes the patient and provider experiences with virtual care across an academic ambulatory hospital in Toronto, Canada during the COVID-19 pandemic. Virtual care, comprised of video and telephone visits, was generally well-received among most patients, with many favoring video visits over telephone visits especially for new patients. However, virtual care was less endorsed among many providers. Furthermore, patients with non-White ethnic backgrounds were less likely to recommend virtual visits. These findings provide important contributions regarding understanding overall patient and provider experiences with virtual care as well as predictors of patient experience. Given the prospect of the hybrid modality of care delivery that includes both virtual and in-person options of care delivery post-COVID-19, future work should aim to develop ways to understand factors to improve patient and provider experiences with virtual care and to assess the impact of virtual modalities on patient outcomes and quality of care.

## Appendix

Multimedia Appendix 1 Patient and provider survey questions.

Multimedia Appendix 2 Supplementary tables.

## Abbreviations

IM: information management
IT: information technology
ON-Marg: Ontario marginalization index
OR: odds ratio
WCH: Women’s College Hospital
WIHV: Women’s College Hospital Institute for Health System Solutions and Virtual Care
