# Supplementary material for: Patient and Provider Experiences With Virtual Care in a Large, Ambulatory Care Hospital in Ontario, Canada During the COVID-19 Pandemic: Observational Study
Source: J Med Internet Res. 2022 Oct 25;24(10):e38604. doi: 10.2196/38604 (PMC9605083; doi:10.2196/38604)
Supplement: Multimedia Appendix 1 [file jmir_v24i10e38604_app1.docx]

**Multimedia Appendix 1**

**Telephone visits survey questions**

1. To date, how many telephone visits have you had with your healthcare team at WCH?

- 1
- 2-3
- 3-4
- 5+

2. Would you have preferred to have had your visit over video instead of by telephone?

- Yes
- No
- Not sure

A. If no, why would you prefer to have your visit over telephone instead of by video? (OPEN TEXT)

B. If yes, why would you have preferred to have your visit by video instead of by telephone? (OPEN TEXT)

3. How much did the telephone visit help you with the health issue for which you needed the appointment?

- Very helpful
- Somewhat helpful
- Neutral
- Not helpful
- Not at all helpful

4. How would you rate your experience with receiving care through a telephone visit compared to an in-person visit?

- Better than an in-person
- Same as an in-person visit
- Worse than an in-person visit
- Not sure

5. How was your ability to access care from your doctor during the telephone visit compared to an in-person visit?

- Better than an in-person
- Same as an in-person
- Worse than an in-person
- Not sure

6. During your most recent video visit, if you had questions to ask your healthcare provider, did you get answers that you could understand?

- Definitely
- For the most part
- Somewhat
- Not at all
- I did not need to ask
- I did not have an opportunity ask

7. During your most recent video visit, how often were you involved as much as you wanted to be in decisions about your care?

- Always
- Usually
- Sometimes
- Never

8. How was your experience receiving care through a telephone visit compared to a video visit?

- I have not previously had a video visit
- Better than a video visit
- Same as a video visit
- Worse than a video visit
- Not sure

If “I have not previously had a video visit” is selected, what is the main reason why you have not had a video visit?

- No one offered the option to have a video visit
- I prefer to have a visit by phone or in-person
- I do not have access to the technology required for a video visit (such as Internet, smartphone or computer)
- I am not comfortable with using technology for video visits
- I tried to have a video visit but it did not work or there were technological challenges
- I am concerned about the privacy or confidentiality of a video visit
- I am physically unable to conduct a video visit (for example because of visual impairments)
- Other (If other is selected OPEN TEXT Please Explain: )

9. What would you have done if you were not able to see your doctor through a telephone visit?

- Walk in clinic
- Emergency department
- See/talk to my family doctor
- Scheduled an in person visit with this doctor
- I would not have sought care at that time

10. Reflecting on your last telephone visit:

A. Did the telephone visit save you time (for example by saving you travel time or arranging care for dependents)?

- Yes
- No
- Unsure

B. Did the telephone visit save you money (for example by cost of transit or parking)?

- Yes
- No
- Unsure

11. Were there any issues that made it hard to participate in the telephone visit?

- Yes
- No

If yes, please describe (free text).

12. How likely are you to recommend the telephone visit to a friend on a scale of 1-10? (1= would not recommend and 10 = would highly recommend)

13. Overall, do you feel you were treated with respect and dignity while you were receiving care from Women’s College Hospital?

- Definitely
- For the most part
- Somewhat
- Not at all

14. Please rate your overall experience with WCH:

| I had a very poor experience |  |  |  |  |  |  |  |  |  | I had a very good experience |
| --- | --- | --- | --- | --- | --- | --- | --- | --- | --- | --- |
| 0 | 1 | 2 | 3 | 4 | 5 | 6 | 7 | 8 | 9 | 10 |

15. How would you rate your overall health?

- Excellent
- Very good
- Good
- Fair
- Poor

16. Would you like the option to continue having telephone visits with your healthcare providers after COVID-19?

- Yes
- No
- Not Sure

17. Do you have any suggestions for how to improve telephone visits you’d like to share with us? (open text)

In order to help us understand if video visits are equitable for patients and suitable to meet the healthcare needs of diverse patients, please answer the following demographic questions. Again, your responses will be kept completely ANONYMOUS to maintain your privacy.

18. [For surveys NOT disseminated on EPIC ONLY ]: How old are you? (enter age)

19. What is your self-identified gender? (Check ALL That apply)

- Female
- Male
- Trans
- Intersex
- Gender Fluid
- Prefer not to answer
- Do not know

20. Do you have any of the following? CHECK ALL THAT APPLY

- Chronic medical illness
- Developmental Disability
- Drug or alcohol dependence
- Learning disability
- Mental Illness
- Physical Disability
- Sensory Disability (eg. hearing or vision loss)
- Other (Please Specify): _____________
- None
- Prefer not to answer
- Do not know

21. Which of the following best describes your racial or ethnic group? CHECK ONE ONLY

- Asian – East (eg. Chinese, Japanese, Korean)
- Asian – South (eg. Indian, Pakistani, Sri Lankan)
- Asian – South East (eg. Malaysian, Filipino, Vietnamese)
- Black – African (eg. Ghanaian ,Kenyan, Somali)
- Black – Caribbean (eg. Barbadian, Jamaican)
- Black – North American (eg. Canadian, American)
- First Nations
- Indian – Caribbean (eg. Guyanese with origins in India)
- Indigenous/Aboriginal not included elsewhere
- Inuit
- Latin America (eg. Argentinean, Chilean, Salvadorian)
- Métis
- Middle-Eastern (eg. Egyptian, Iranian, Lebanese)
- White – European (eg. English, Italian, Portuguese, Russian)
- White – North American (eg. Canadian, American)
- Mixed heritage (Please Specify): __________________
- Other(s) (Please Specify): ___________________
- Prefer not to answer
- Do not know

22. What was your total family income before taxes last year? CHECK ONE ONLY

- $0 - $29 999
- $30 000 - $59 999
- $60 000 - $89 999
- $90 000 - $119 999
- $120 000 - $149 999
- $150 000 or more
- Prefer not to answer
- Do not know

23. How would you rate your ability to speak and understand English? CHECK ONE ONLY

- Very well
- Well
- Not well
- Not at all
- Unsure
- Prefer not to answer
- Do not know

24. [For surveys NOT disseminated on EPIC ONLY]: Please provide us with the first 3 letters of your postal code (this data will only be used to help us understand if there are differences in virtual care utilization based on geography/area of residence): _ _ _

**Appendix B: Video visit survey questions**

1. To date, how many video visits have you had with your healthcare team at WCH?

- 1
- 2-3
- 3-4
- 5+

If 1, then question 3

If >1, then skip to question 5

2. Did you look at the training information prior to conducting your first video visit?

- Yes
- No

If Yes, please include any additional feedback about the training for video visits. (free text)

3. Right before your appointment, how confident did you feel in your ability to participate in a video visit?

- Very confident
- Somewhat confident
- Neutral
- Not confident
- Not at all confident

4. To what degree did the video visit help you with the health issue for which you needed the appointment?

- Very helpful
- Somewhat helpful
- Neutral
- Not helpful
- Not at all helpful

5. On average, compared to an in-office visit how was your experience with receiving care through a video visit?

- Better than an in-person
- Same as an in-person visit
- Worse than an in-person visit
- Not sure

6. On average, compared to an in-office visit how did the video visit impact your ability to access care from you doctor?

- Better than an in-person
- Same as an in-person
- Worse than an in-person
- Not sure

7. On average, compared to a telephone visit how was your experience receiving care through a video visit?

- I have not previously had a telephone visit
- Better than a telephone visit
- Same as a telephone visit
- Worse than a telephone visit
- Not sure

8. What would you have done if you were not able to see your doctor through a video visit?

- Walk in clinic
- Emergency department
- See/talk to my family doctor
- Scheduled an in person visit with this doctor
- I would not have sought care at that time

9. Reflecting on your last video visit, did the video visit save you time (for example by saving you travel time or arranging care for dependents)?

- Yes
- No
- Unsure

10. Reflecting on your last video visit, did the video visit save you money (for example by cost of transit or parking)?

- Yes
- No
- Unsure

11. Were there any issues with technology that made it difficult to participate in a video visit?

- Yes
- No

If yes, what issues made it hard to participate? (free text)

12. How likely are you to recommend the virtual visits to a friend on a scale of 1-10? (1 = would not recommend and 10 = would highly recommend)

13. How do you rate your comfort with using technology in your day-to-day life?

- Excellent
- Very good
- Good
- Fair
- Poor

14. How would you rate your overall health?

- Excellent
- Very good
- Good
- Fair
- Poor

In order to help us understand if video visits are equitable for patients with diverse characteristics, please answer the following four demographic questions. Again, your responses will be kept completely ANONYMOUS to maintain your privacy.

15. What is your self-identified gender? (check ALL that apply)

- Female
- Male
- Trans
- Intersex
- Gender fluid
- Prefer not to answer
- Do not know

16. How would you rate your ability to speak and understand English? CHECK ONE ONLY

- Very well
- Well
- Not well
- Not at all
- Unsure
- Prefer not to answer
- Do not know

17. Which of the following best describes your racial or ethnic group? CHECK ONE ONLY

- Asian – East (eg. Chinese, Japanese, Korean)
- Asian – South (eg. Indian, Pakistani, Sri Lankan)
- Asian – South East (eg. Malaysian, Filipino, Vietnamese)
- Black – African (eg. Ghanaian ,Kenyan, Somali)
- Black – Caribbean (eg. Barbadian, Jamaican)
- Black – North American (eg. Canadian, American)
- First Nations
- Indian – Caribbean (eg. Guyanese with origins in India)
- Indigenous/Aboriginal not included elsewhere
- Inuit
- Latin America (eg. Argentinean, Chilean, Salvadorian)
- Métis
- Middle-Eastern (eg. Egyptian, Iranian, Lebanese)
- White – European (eg. English, Italian, Portuguese, Russian)
- White – North American (eg. Canadian, American)
- Mixed heritage (Please Specify): __________________
- Other(s) (Please Specify): ___________________
- Prefer not to answer
- Do not know

18. What was your total family income before taxes last year? CHECK ONE ONLY

- $0 - $29 999
- $30 000 - $59 999
- $60 000 - $89 999
- $90 000 - $119 999
- $120 000 - $149 999
- $150 000 or more
- Prefer not to answer
- Do not know

19. Would you like the option to continue having video visits with your healthcare providers after COVID-19?

- Yes
- No
- Not Sure

20. Any suggestions for how to improve video visits for patient care? (open text)
