# Supplementary material for: Patient and Provider Experiences With Virtual Care in a Large, Ambulatory Care Hospital in Ontario, Canada During the COVID-19 Pandemic: Observational Study
Source: J Med Internet Res. 2022 Oct 25;24(10):e38604. doi: 10.2196/38604 (PMC9605083; doi:10.2196/38604)
Supplement: Multimedia Appendix 2 [file jmir_v24i10e38604_app2.docx]

**Multimedia Appendix 2**

**Table S1.** New patients only – baseline demographic characteristics of survey respondents.

| Characteristic | Video visit survey respondents (N=109)^a^ | % among non-missing respondents | Phone visit survey respondents (N=21)^b^ | % among non-missing respondents | *P* value |
| --- | --- | --- | --- | --- | --- |
| **Gender, n(%)** |  |  |  |  | 0.244 |
| Female | 83 (76.2%) | 76.2% | 19 (90.5%) | 90.5% |  |
| Male | 26 (23.9%) | 23.9% | 2 (9.5%) | 9.5% |  |
| **Age, mean (SD)** | 41.3 (15.3) | - | 50.5 (17.5) | - | 0.015* |
| **Total family income in previous year^c^, n(%)** |  |  |  |  | 0.746 |
| $0 to $29,999 | 6 (5.5%) | 7.8% | 1 (4.8%) | 4.8% |  |
| $30,000 to $59,999 | 7 (6.4%) | 9.1% | 1 (4.8%) | 4.8% |  |
| $60,000 to $89,999 | 7 (6.4%) | 9.1% | 2 (9.5%) | 9.5% |  |
| $90,000 to $119,000 | 7 (6.4%) | 9.1% | 3 (14.3%) | 14.3% |  |
| $120,000 to $149,000 | 3 (2.8%) | 3.9% | 1 (4.8%) | 4.8% |  |
| $150,000 or more | 18 (16.5%) | 23.4% | 4 (19.1%) | 19.1% |  |
| Do not know | 5 (4.6%) | 6.5% | 0 (0%) | 0% |  |
| Prefer not to answer | 24 (22.0%) | 31.2% | 9 (42.9%) | 42.9% |  |
| Missing | 32 (29.4%) | - | 0 (0%) | - |  |
| **Ethnic group^d^, n(%)** |  |  |  |  | 1.000 |
| White | 59 (54.1%) | 72.8% | 15 (71.4%) | 75.0% |  |
| Asian | 6 (5.5%) | 7.4% | 2 (9.5%) | 10.0% |  |
| Black | 3 (2.8%) | 3.7% | 1 (4.8%) | 5.0% |  |
| Latin American | 4 (3.7%) | 4.9% | 0 (0%) | 0% |  |
| Indigenous | 1 (0.9%) | 1.2% | 0 (0%) | 0% |  |
| Middle Eastern | 1 (0.9%) | 1.2% | 0 (0%) | 0% |  |
| Mixed heritage/Other(s) | 2 (1.8%) | 2.5% | 1 (4.8%) | 5.0% |  |
| Prefer not to answer | 5 (4.6%) | 6.2% | 1 (4.8%) | 5.0% |  |
| Missing | 28 (25.7%) | - | 1 (4.8%) | - |  |
| **English ability, n(%)** |  |  |  |  | 0.493 |
| Very well | 77 (70.6%) | 95.1% | 19 (90.5%) | 90.5% |  |
| Well | 3 (2.8%) | 3.7% | 1 (4.8%) | 4.8% |  |
| Not well | 1 (0.9%) | 1.2% | 1 (4.8%) | 4.8% |  |
| Missing | 28 (25.7%) | - | 0 (0%) | - |  |
| **Ontario marginalization index, n(%)** |  |  |  |  | 1.000 |
| Marginalized | 12 (11.0%) | 11.8% | 2 (9.5%) | 10.0% |  |
| Not marginalized | 90 (82.6%) | 88.2% | 18 (85.7%) | 90.0% |  |
| Missing | 7 (6.4%) | - | 1 (4.8%) | - |  |

^a^ N=109 is number of unique patients who responded. N=117 is number of video survey responses received. Same patient may be counted multiple times.
^b^ N=21 is number of unique patients who responded. N=22 is number of phone survey responses received. Same patient may be counted multiple times.
^c^ P-value compares <$90,000 versus $90,000+
^d^ P-value compares White versus non-White
**Statistically significant*

**Table S2.** Top 10 clinical departments for telephone visits.

| **Clinical department** | **N(%) of total telephone visit surveys (N=209)** |
| --- | --- |
| General endocrinology | 98 (46.9%) |
| Centre for headache | 24 (11.5%) |
| Reproductive life stages program | 12 (5.7%) |
| Trauma therapy | 10 (4.8%) |
| Cardiology | 10 (4.8%) |
| Addictions medicine | 4 (1.9%) |
| Diabetes program | 4 (1.9%) |
| Mental health in medicine and general psychiatry | 3 (1.4%) |
| Osteoporosis program | 3 (1.4%) |
| Respirology program | 3 (1.4%) |

**Table S3.** Top 10 clinical departments for video visits.

| **Clinical department** | **N(%) of total video visit surveys (N=517)** |
| --- | --- |
| Mental health | 137 (26.5%) |
| COVID care at home | 69 (13.4%) |
| Reproductive life stages program | 62 (12.0%) |
| Centre for headache | 38 (7.4%) |
| Cardiology | 34 (6.6%) |
| Rheumatology | 34 (6.6%) |
| Mental health in medicine and general psychiatry | 20 (3.9%) |
| Social work | 14 (2.7%) |
| Physiotherapy | 12 (2.3%) |
| Brief psychotherapy | 12 (2.3%) |
| General surgery | 12 (2.3%) |

**Table S4.** New patients only – video and phone survey responses.

| **Question** | **Video visit responses**  **(N=117)** | **Phone visit responses**  **(N=22)** | ***P-*value** |
| --- | --- | --- | --- |
| **To what degree did the video or phone visit help you with the health issue for which you needed the appointment?** |  |  | 0.017* |
| Not at all helpful | 0 (0%) | 1 (4.6%) |  |
| Not helpful | 0 (0%) | 1 (4.6%) |  |
| Neutral | 9 (7.7%) | 3 (13.6%) |  |
| Somewhat helpful | 26 (22.2%) | 3 (13.6%) |  |
| Very helpful | 81 (69.2%) | 14 (63.6%) |  |
| - | 1 (0.9%) | 0 (0%) |  |
| **What would you have done if you were not able to see your doctor through a video or phone visit?^a^** |  |  | 0.522 |
| Walk-in clinic | 5 (4.3%) | 0 (0%) |  |
| Emergency department | 5 (4.3%) | 1 (4.6%) |  |
| See/talk to my family doctor | 33 (28.2%) | 6 (27.3%) |  |
| Scheduled an in-person visit with this doctor | 45 (38.5%) | 11 (50.0%) |  |
| I would not have sought care at that time | 41 (35.0%) | 4 (18.2%) |  |
| - | - | 0 (0%) |  |
| **How likely are you to recommend video or phone visits to a friend on a scale of 1-10? (1 = would not recommend and 10 = would highly recommend)** |  |  | NA |
| 1 | 0 (0%) | 0 (0%) |  |
| 2 | 0 (0%) | 1 (4.6%) |  |
| 3 | 1 (0.9%) | 0 (0%) |  |
| 4 | 2 (1.7%) | 0 (0%) |  |
| 5 | 6 (5.1%) | 1 (4.6%) |  |
| 6 | 5 (4.3%) | 3 (13.6%) |  |
| 7 | 9 (7.7%) | 3 (13.6%) |  |
| 8 | 17 (14.5%) | 4 (18.2%) |  |
| 9 | 18 (15.4%) | 1 (4.6%) |  |
| 10 | 54 (46.2%) | 7 (31.8%) |  |
| - | 5 (4.3%) | 2 (9.1%) |  |
| **Net promoter score** | **51.8%** | **15.0%** |  |
| **Would you like the option to continue having virtual visits with your healthcare providers after COVID-19?** |  |  | <.001* |
| No | 2 (1.7%) | 5 (22.7%) |  |
| Not sure | 22 (18.8%) | 3 (13.6%) |  |
| Yes | 92 (78.6%) | 14 (63.6%) |  |
| Missing | 1 (0.9%) | 0 (0%) |  |

^a^Multi-select question for video visit survey
**Statistically significant*

**Table S5.** Average recommendation slider score by ethnicity (addition to regression output).

|  | **Video, mean(SD)** | **Phone, mean(SD)** |
| --- | --- | --- |
| **White** | 8.8 (1.6) | 8.5 (2.1) |
| **Asian** | 9.7 (0.5) | 8.3 (1.5) |
| **Black** | 9.5 (0.6) | 8.4 (1.5) |
| **Latin American** | 8 (2.7) | 7.7 (2.3) |
| **Indigenous** | 8 (-) | 8.6 (0.9) |
| **Middle Eastern** | 7.5 (3.5) | 9.3 (1.2) |
| **Mixed heritage/Other(s)** | 8 (1.7) | 7.3 (2.1) |
| **Prefer not to answer** | 9.6 (0.9) | 7.4 (2.4) |
| **Missing** | 8.9 (1.6) | 8 (2.5) |
